# In Vitro Simulation and In Vivo Assessment of Tooth Wear: A Meta-Analysis of In Vitro and Clinical Research

**DOI:** 10.3390/ma12213575

**Published:** 2019-10-31

**Authors:** Despina Koletsi, Anna Iliadi, Theodore Eliades, George Eliades

**Affiliations:** 1 Clinic of Orthodontics and Pediatric Dentistry, Center of Dental Medicine, University of Zurich, 8032 Zurich, Switzerland; d.koletsi@gmail.com; 2 Department of Biomaterials, School of Dentistry, National and Kapodistrian University of Athens, 11527 Athens, Greece; annaeliades@gmail.com (A.I.); geliad@dent.uoa.gr (G.E.)

**Keywords:** tooth wear, enamel wear, in vitro wear simulation, tooth loading, composite, porcelain, ceramic, zirconia, lithium disilicate, systematic review, meta-analysis

## Abstract

Tooth wear may be described as a side-effect of occlusal forces that may be further induced by the common use of contemporary prosthetic materials in practice. The purpose of this systematic review was to appraise existing evidence on enamel wear from both in vitro and clinical research and explore whether evidence from these study designs lies on the same direction. Five databases of published and unpublished research were searched without limitations in August 2019 and study selection criteria included in vitro and clinical research on enamel tooth wear. Study selection, data extraction, and risk of bias assessment were done independently and in duplicate. Random effects meta-analyses of standardized mean differences (SMDs) or weighted mean differences (WMDs) with 95% confidence intervals (CIs) were conducted while a Monte Carlo permutation test for meta-regression on the exploration of the effect of the study design on the reported outcomes was planned. A total of 27 studies (23 in vitro and 4 clinical) were eligible while 12 contributed to meta-analyses. Overall, some concerns were raised for the quality of the existing evidence and the potential for risk of bias. Enamel wear (mm) of antagonist teeth was more pronounced when opposed to conventional porcelain compared to machinable ceramics (SMD = 2.18; 95%CIs: 1.34, 3.02; *p* < 0.001). Polished zirconia resulted in decreased volumetric enamel wear (mm^3^) of opposing teeth compared to pure natural enamel (SMD = –1.06; 95%CIs: –1.73, –0.39; *p* = 0.002). Monolithic zirconia showed evidence of enhanced potential for antagonist wear (μm) compared to natural teeth (WMD = 107.38; 95%CIs: 30.46, 184.30; *p* = 0.01). Study design did not reveal an effect on the tooth wear outcome for the latter comparison when both clinical and in vitro studies were considered (three studies; Monte Carlo test, *p* = 0.66). In conclusion, there is an overriding need for additional evidence from clinical research to substantiate the findings from the already existing laboratory simulation studies.

## 1. Introduction

Tooth wear is a multi-faceted common dental condition, which constitutes a variable amount of tooth substance loss not related to cariogenic conditions. Wear may appear as a result of the effect of acid-containing solutions [1], mechanical loading and related forces generated by masticatory function within a tooth-to-tooth or tooth-to-restorative material contact interface [2], or else through abrasive effects of intermediate factors (i.e., abrasive particles) [3]. Tooth wear has been linked to several alarming or clinically disturbing conditions for the patient, namely loss of vertical dimension, disorders related to the temporomandibular joint, aesthetic considerations, or hypersensitivity [4,5]. The amount of wear is therefore evidently a significant determinant of the severity of clinical manifestations.

Currently, different types of ceramic materials as well as composite resins are used for prosthetic restorations after an increasing demand for nonmetallic dental prostheses. Massive use of dental restorations in clinical practice, through a range of different/ novel materials, such as a number of materials based on metal alloys, particle-reinforced polymers, and ceramics, have increased awareness regarding maintenance of antagonist natural tooth integrity [6,7]. Recently, advances in the development of prosthetic dental materials used as crown or bridge tooth substitutes have introduced additional considerations regarding their contact interface and possible detrimental effects on sound enamel structure. Ceramic restorations, although presenting unanimous advantages regarding their clinical performance, biotolerance, and aesthetic appearance, may still constitute materials of increased hardness, being resistant to fractures. As such, opposing enamel tooth wear might be a common side-effect [8,9]. Composite restorations may present antagonist tooth wear considerations as well. This is mainly dependent on the chemical composition of the matrix and the quality of the filler components [6,10]. It has been claimed that the inclusion of nano- or sub-micron particle-sized fillers in the composite resin may make the material more resistant to wear, thus increasing the propensity for antagonist enamel wear [11].

The dynamic interface of natural tooth enamel and prosthetic dental materials is an area subjected to certain stress conditions when natural dentition is compromised, especially following increasing years of functional capacity of the masticatory system [12]. Although most restorative materials resemble enamel, they do not necessarily have similar characteristics in terms of surface roughness, fracture toughness, and microstructural features. At a microstructural level, enamel consists of mineral rods separated by thin protein-rich sheaths [13]. The nanostructure of each rod consists of hydroxyapatite crystals separated by a thin protein layer. When axial contacts with abrasive particles occur, the enamel is affected in both micro- and nano-structural levels. Initially, a defect near the surface is caused by particle micro-indentation, followed by the apparent demineralization of an area deeper below the contact point. The shape of the demineralized region resembles a quasi-plastic zone observed in coarse grain ceramics [14]. 

In view of the above, research has been directed towards a more holistic evaluation of the clinical performance of various prosthetic materials used in vivo, with a more definite evaluation of possible detrimental effects on the antagonist enamel. Numerous studies have been performed in vitro, in an attempt to elucidate the wear of prosthetic restorations and opposing enamel. The preponderance of these studies have used extracted teeth specimens against prosthetic restoration materials and in vitro methods for the simulation of the masticatory cycle and chewing function [15,16,17]. Although large-scale clinical research might have been more desirable to fully integrate the perceived wear of enamel within the oral cavity equilibrium, it is indisputably more difficult to control for individual variation patterns of stomatognathic function. Thus, a limited number of clinical studies on this topic exist, compared to their in vitro design counterparts [2,18,19]. The consistency of the findings from in vitro and clinical studies is yet to be assessed and is likely to provide useful insights to the applicability and extrapolation of laboratory findings to clinical practice [8]. 

Therefore, the aim of this study was to systematically collect and appraise the evidence from both in vitro and clinical research on enamel tooth wear as opposed to different prosthetic restoration materials. The principal null hypothesis of this systematic review was that there was no difference in the amount of antagonist enamel tooth wear as opposed to a wide range of prosthetic restorative materials. Our further null hypothesis was that evidence from in vitro simulation and in vivo clinical assessment of tooth wear is on the same direction. 

## 2. Materials and Methods 

### 2.1. Protocol Registration and Reporting

This systematic review was a priori registered at the International Prospective Register of Systematic Reviews PROSPERO (registration identifier to be released), as well as at the Open Science Framework (https://osf.io/dja8h/). For reporting, the Preferred Reporting Items for Systematic Reviews and Meta-Analyses (PRISMA) [20,21] were followed. 

### 2.2. Eligibility Criteria

The following inclusion/exclusion criteria were applied for this systematic review according to the Participants, Intervention, Comparator, Outcomes (PICO) guidance [20,21], modified accordingly for the in vitro research design types:

- Study design: Clinical studies comprising randomized controlled (RCTs) studies or controlled clinical trials as well as in vitro simulation studies with extracted teeth as control groups;
- Participants: For clinical studies, participants of both sexes and of any age range were included. Prosthetic–enamel or enamel–enamel tooth interfaces were considered. For in vitro research, intact extracted human teeth of any type were considered eligible. Enamel laboratory-produced surface or cut enamel, enamel slabs, or plates were excluded;
- Intervention: Intact enamel of teeth;
- Comparator: Any type of prosthetic material or enamel antagonist; and
- Outcome(s): Enamel tooth wear was the primary outcome considered. This included assessment methods within the range of but not confined to volume loss, vertical wear, height loss, and depth wear. Roughness changes in enamel surface were not considered.

### 2.3. Search Strategy

An electronic search was employed within five major databases for both published and unpublished research. Medline via PubMed, Cochrane Central Register of Controlled Trials (CENTRAL), ClinicalTrials.gov (www.clinicaltrials.gov), National Research Register (ISRCTN: www.controlled-trials.com), and Open Grey were accessed between 28 July and 4 August 2019 to identify relevant research papers or study protocols (Appendix A). Representative keywords used included “tooth wear”, “enamel wear”, “in vitro simulation”, “chewing”, “mastication cycle”, and others. A full-search strategy for Medline is presented in Appendix A. Screening for eligible titles, abstracts, and full texts was initially implemented by one reviewer (DK) and confirmed by a second (AI) while any disagreements were discussed and settled through consultation with a third author (TE).

### 2.4. Data Extraction

Data extraction was performed independently by one reviewer (DK) and reviewed by a second (AI) and entailed extraction of relevant information on standardized piloted forms. Information on year of publication, origin, study design, sample size, method of analysis, interventions/comparators, and outcomes, as well as loading and simulation cycles for the in vitro studies were recorded. The reviewers were not blinded to the study title and authorship. 

### 2.5. Risk of Bias Assessment within Individual Studies

For the included clinical studies, the updated Cochrane risk of bias tool (RoB 2.0) [22] was used to evaluate the perceived risk of bias within the randomized studies while the ROBINS-I tool [23] was used to assess the risk of bias on non-randomized clinical studies. Likewise, for in vitro research and as no pre-determined guidelines to assess the risk of bias exist, a modification of the Cochrane tool was implemented with an attempt to incorporate specific important elements that would help identify the presence of potential bias. These include selection bias, performance bias, attrition bias, and reporting issues. Again, the assessment of specific elements to detect potential bias was performed by one reviewer (AI) and also duplicated by a second (DK). Any discrepancies were resolved through consultation with a third reviewer (TE).

### 2.6. Summary Measures and Data Synthesis

Before any decision to pool data from individual studies was made, clinical heterogeneity was assessed in terms of individual trials or laboratory settings (i.e., simulation cycles, loading protocols), eligibility criteria, or data collection methods. Subsequently, statistical heterogeneity was initially examined through visual inspection of the confidence intervals (CIs) of the estimated intervention effects on the forest plots. In addition, a formal chi-square test was also conducted to assess substantial statistical heterogeneity, as indicated by a *p*-value below the level of 10% for the test (*p* < 0.10) [24]. The *I*^2^ test for homogeneity was undertaken as well. 

Random effects meta-analyses were conducted as they were considered more appropriate to reflect the expected heterogeneity and variations in laboratory settings and simulation-related conditions, or between patient variability for clinical studies. As continuous outcomes were expected overall, treatment effects were calculated through pooled standardized mean differences (SMDs) or weighted mean differences (WMDs) with associated 95% confidence intervals (95% CIs) and prediction intervals where possible (at least three studies needed). The results from clinical and in vitro studies were not pooled together. 

### 2.7. Risk of Bias Across Studies

If more than 10 studies were included in meta-analyses, publication bias was explored through standard funnel plots [25] and Egger’s regression test [26].

### 2.8. Additional Analyses

Sensitivity analyses were predetermined to explore and isolate the effect of studies with low risk of bias on the pooled treatment effect if both low and higher risk of bias studies were finally included. In addition, a Monte Carlo permutation test for meta-regression was planned if a sufficient number of studies that belonged to both study designs (in vitro, clinical) were retrieved, in order to inspect the effect of the design on the reported effect estimate for a specific outcome. 

All analyses were undertaken in Stata version 15.1 software (StataCorp, College Station, Texas, USA). 

## 3. Results

### 3.1. Search Details

**Figure 1:**
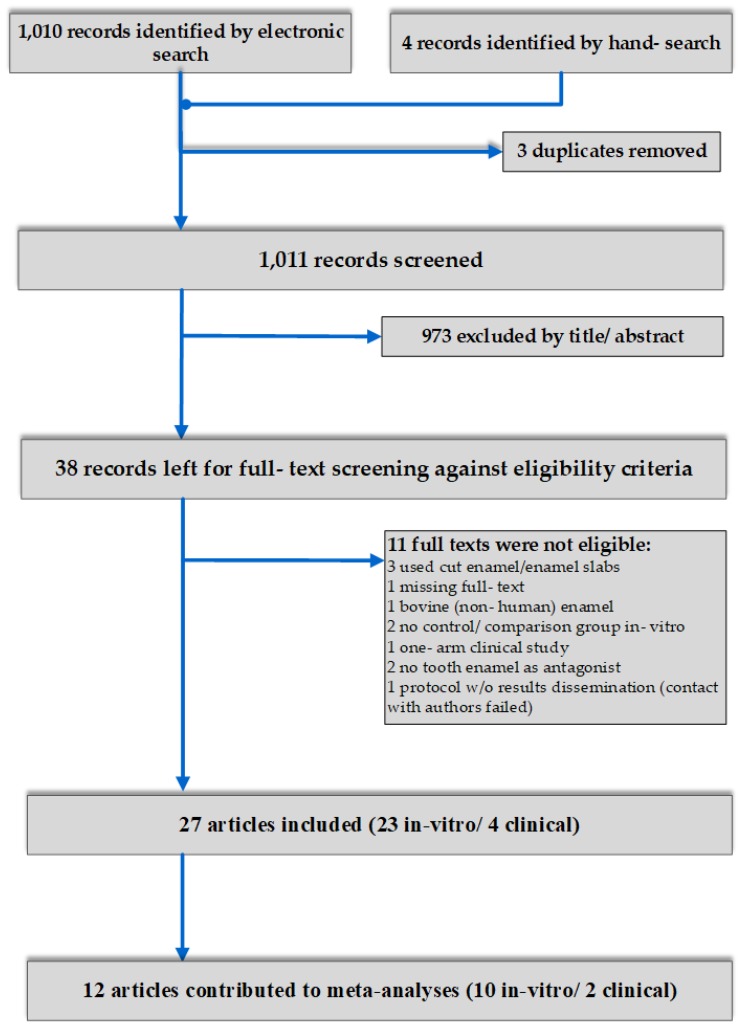
Flow diagram of the article selection.

In total, 1014 studies were initially retrieved after electronic and hand searching. After duplicate exclusion, and title and abstract screening, 38 records were left for full-text evaluation. Eleven papers were excluded due to reasons related to our pre-defined eligibility criteria, leaving a final number of 27 studies included in the systematic review. Of those, a preponderance of studies belonged to laboratory simulation studies (n = 23) of tooth wear, while only four were clinical studies in real-time conditions (Figure 1).

### 3.2. Study Design and Characteristics

Of the four clinical trials eligible for inclusion, three were randomized controlled trials while the remaining one was a non-randomized prospective clinical study. In vitro studies were conducted within years 1997 to 2019 while the four trials presented a tighter and most recent publication record and were published between 2014 and 2019. The vast majority were conducted outside European or American countries (17/27, 63%), with many representatives from Asia (Korea or India). Six studies originated from European countries (Italy, Germany, Switzerland) and the remaining four were from the United States or Brazil (Table S1).

Sample sizes for the in vitro studies ranged from 5 to 100 human premolars or molars, evidently broken down to a decreased number of teeth per comparison due to the necessity of forming different groups of small samples of the assessed numerous prosthetic materials to be examined against human enamel. As such, sample sizes per group ranged from 5 to 30 teeth. The most widely studied prosthetic material in terms of wear of the enamel of the antagonist tooth was zirconia, across different (surface) material treatments. Polished, glazed, combinations of polishing and glazing, and adjusted or unadjusted monolithic zirconia were primarily studied. Other material samples were also examined, such as lithium disilicate, feldspathic porcelain, low fusing hydrothermal ceramic, or composite materials. As for the simulation conditions, different loading cycles within the wide range of 5000 to 1,200,000 cycles were described. The loading conditions currently reported correspond to a range of nearly 10 to 340 N, while most studies reported the use of a load of nearly 50 N for clinical simulation. The latter represents the most frequent load level used for a masticatory/chewing simulator, while this numerical loading value in combination with a range of cycles of approximately 200,000 to 300,000 has been described as a potential simulation of a 1-year in vivo chewing/mastication. However, other combinations have also been reported (Table S1). The preponderance of tooth wear assessment methods constituted 3D laser or optical profilometry, laser scanner, or reflex microscope. 

For the clinical studies assessing tooth wear in vivo, the three RCTs and the sole prospective non-randomized trial included a total of 10 to 30 patients, according to the individual studies, with 6 to 17 teeth contributing per group under examination. Effects of monolithic zirconia, polished zirconia, metal ceramic, and pure opposing enamel to antagonist tooth wear were studied. The duration period for assessment ranged from six months to one year, while methods for outcome assessment (i.e., enamel wear) were confined to a 3D laser scanner or desktop scanner for casts (Table S1). 

### 3.3. Risk of Bias within Studies

**Figure 2:**
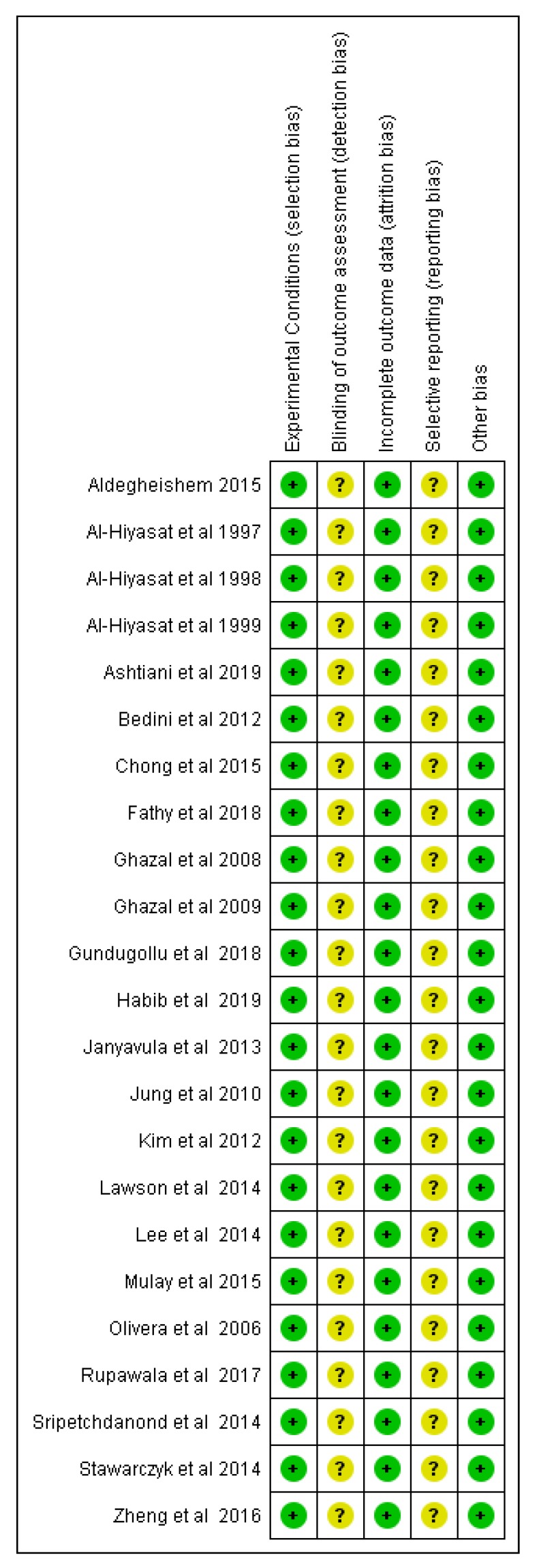
Risk of bias summary outlining judgment of risk of bias items for each of the included in vitro studies (n = 23). The plus sign indicates low risk of bias; the circle with a question mark indicates an unclear risk of bias.

The risk of bias of the in vitro studies was explored using a modified version of the Cochrane risk of bias tool. In all, the experimental conditions were explicitly described for all reported material testing, while no losses or destruction of specimens was documented that would allow for the potential of attrition bias. Blinding of the outcome assessors was not described by any of the included studies and this would raise some concerns regarding the unbiased judgement of the reported outcomes, mainly pertaining to enamel wear. Last, the reported findings of the studies were adequately supported by the respective methodologies described in the publication, although no study reported a pre-registration of the project protocol (Figure 2). 

For the clinical studies, the three RCTs [2,18,19] were rated on the perceived risk of bias using the RoB 2.0 tool [22]. For two of them [2,19], concealment of the allocation sequence was not reported or implied, raising concerns about the potential for selection bias. Moreover, none reported masking of the outcome assessor, raising concerns whether knowledge of the intervention could potentially influence the assessment of the outcome. There was also no information about pre-registration of trial protocols or any pre-specified analysis plan by the researchers of each RCT finalized before any data became available for analysis (Table 1, Table S2). The sole prospective clinical trial included in this review [27] was rated according to the ROBINS-I tool [23]. Again, issues of selection bias due to confounding stemming from potential prognostic factors that were not controlled for appeared. In the same direction as the RCTs, some concerns were also noted for the potential selection of the reported results, as no registered trial protocol existed (Table 2, Table S3).

### 3.4. Effects of Interventions, Meta-Analyses, and Additional Analyses

In total, 13 quantitative syntheses were eligible, while all consisted of a maximum of two contributing studies. Twelve comparisons were possible for in vitro research, while only one for clinical (Table 3). In essence, 10 studies of in vitro research and two of clinical contributed to the syntheses. All related outcomes pertained to enamel wear as antagonist to prosthetic material or natural teeth, mainly expressed as vertical wear (in μm or mm) or volumetric wear (mm^3^).

Based on in vitro research, it was evident that a great number of comparisons between prosthetic material specimens and opposing natural tooth enamel were performed. Following the results of the formulated quantitative syntheses with at least two studies included, we recorded increased vertical tooth wear (mm) of the opposing enamel when conventional porcelain was compared to machinable ceramics (SMD = 2.18; 95%CIs: 1.34, 3.02; *p* < 0.001). Likewise, when polished zirconia was compared to pure natural enamel, it was evident that volumetric enamel wear (mm^3^) of the antagonist was increased in the natural teeth group (SMD = 1.06; 95%CIs: 0.39, 1.73; *p* = 0.002; Table 3). Both aforementioned comparisons showed low levels of heterogeneity, respectively, at 8.4% and 0%. Additional comparisons pertained the assessment of opposing enamel wear induced by zirconia with different types of surface treatment, such as polished-to-glazed or polished-to-polished and glazed zirconia, monolithic zirconia-to-lithium disilicate, composite resin, low-fusing hydrothermal ceramic-to-conventional porcelain, or machinable ceramics. No differences were detected for any of those comparisons with respect to different treatment outcomes pertaining to enamel wear, while highly variable levels of heterogeneity were inspected, being presented within the range of 0% to 95.4%. Other individual study considerations, not included in the meta-analyses, pertained to the comparisons of specific zirconia materials constituting different surface roughness and their effect on antagonist enamel. According to these, a smoother material surface induced decreased enamel wear [28]. 

**Figure 3:**
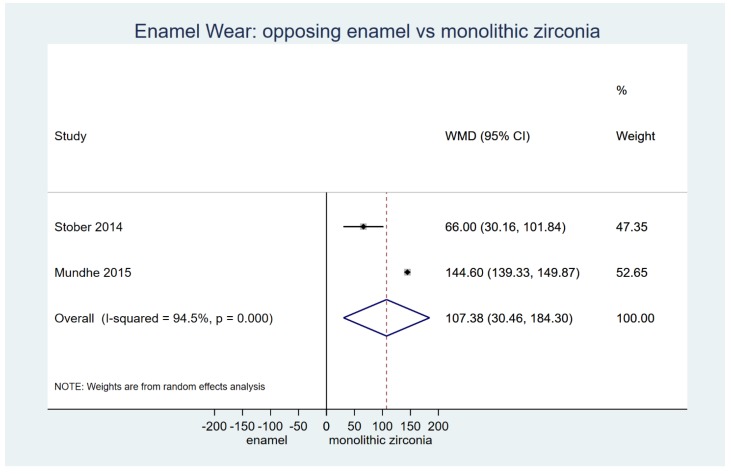
Random effects meta-analysis to explore antagonist enamel wear (μm) for monolithic zirconia versus natural enamel in clinical studies.

Based on clinical research from one RCT [2] and one prospective non-randomized study [27], a strong effect of monolithic zirconia on antagonist wear was detected when compared to natural teeth counterparts (two studies on vertical wear μm: WMD = 107.38; 95%CIs: 30.46, 184.30; *p* < 0.001), for a clinically relevant time interval of 6 to 12 months (Table 3; Figure 3). Furthermore, when exploring the findings from individual clinical studies that could not be mathematically combined, it was evident that metal-ceramic crowns showed an increased capability of producing antagonist enamel wear than monolithic zirconia (*p* < 0.001) [2]. On the contrary, more recent findings from a 1-year RCT on the behavior of monolithic ceramic versus metal ceramic on opposing enamel demonstrated no significant differences between the two materials [18]. However, the latter study lacked formal and precise information on data variability, while any attempt to contact the authors was unsuccessful. Last, a randomized study that examined the effect of different types of polished translucent zirconia crowns showed evidence of increased antagonist enamel wear in line with the increased surface roughness of the material and phase transformation after clinical use [19]. 

Pre-planned analyses to explore publication bias and small study effects were ultimately not performed due to limited data and the number of studies for the quantitative syntheses. Sensitivity analyses to substantiate the robustness of our findings solely based on low risk of bias studies was also not applicable. Last, a meta-regression based on three studies did not reveal an effect of study design on the tooth wear outcome for the comparison between monolithic zirconia and natural enamel (Monte Carlo test, *p* = 0.66).

## 4. Discussion

### 4.1. Summary of the Evidence

The present systematic review with meta-analyses revealed a wide variety of laboratory simulation studies, following masticatory and chewing cycles, on the effect of contemporary prosthetic materials on enamel tooth wear in vitro. Likewise, effectively fewer in vivo clinical studies on the topic were retrieved and were simultaneously assessed. 

The primary null hypothesis was rejected at a global level. On one side, clinical research showed evidence of increased wear of the enamel antagonist when opposed to monolithic zirconia, following comparison to natural teeth interface. On the other side, conventional ceramics were more prone to induce wear compared to machinable ceramic crowns, while surface treatment of materials through polishing methods, at least in vitro, demonstrated the capacity of the material to pertain low antagonist wear potential, even lower than human natural enamel. Effectively, when examining the effect on tooth wear from the perspective of the study design and especially with regard to monolithic zirconia, no difference was ultimately detected and the null hypothesis could not be rejected. However, this finding should probably be interpreted with caution, as a considerably low number of studies contributed to this exploratory analysis. Furthermore, if one inspects carefully the treatment effects from each one of the contributing studies [2,11,27], it is evident that the RCT bears the most pronounced effect, attesting to the capacity of monolithic zirconia to produce enamel wear [2], followed by a less evident but significant effect in the same direction stemming from the prospective non-randomized study [27]; however, the in vitro simulation study appeared to fall behind those two [11].

Overall, enamel as well as ceramic restorations wear through a similar process, whereas metal and composite restorations wear through an adhesion mechanism [29]. Gold alloys are still considered the gold standard since they resemble both enamel function and wear characteristics. Gold restorations are proven to cause less enamel wear than ceramic restorations [30]. Ceramic restorations with a smooth surface have demonstrated less tooth wear compared to rough surface porcelain. It has been claimed that increased levels of hardness of the material cannot predict or substantiate a higher wear potential against the enamel surface [3,19]. Alternative factors, such as the surface roughness of the materials, appear to possess a significant role in the determination of their capacity for antagonist enamel wear [8,19]. As such, techniques used for surface treatment of the prosthetic restorative materials, such as polishing or glazing, or intraoral aging may alter surface roughness levels and bear an effect on their wear potential [28,31]. Porcelain surface treatment (rough, polished, or glazed) has been identified as an important factor that determines enamel wear caused by ceramics. A number of studies have determined that polished ceramic surfaces cause less enamel wear than glazed surfaces [32]. In addition, the fracture toughness or flexural strength of conventional porcelain restorations may set the conditions for localized stress areas in the enamel surface that may lead to increased levels of wear. This is particularly important for feldspathic porcelain, where crystalline particles may appear after stress conditions within the material and act as an abrasive factor for the antagonist [33,34]. Conventional composite materials wear through a process on which the resin matrix is worn away and the filler particles are lost [10]. As a result, composite restorations are often excessively worn by enamel, porcelain, and other restorative materials [28]. However, highly filled posterior composites may abrade human enamel differently due to the altered size, hardness, and content of the filler particles [10]. Recently, monolithic zirconia has been used for posterior restorations because of its high fracture resistance. Zirconia restorations seem to cause less enamel wear when compared to feldspathic porcelains as well as heat-pressed lithium disilicate glass ceramics. Moreover, ceramic antagonists have been reported to experience greater volumetric loss when compared to zirconia restorations, since the complex interactions between glassy and crystalline phases in their microstructure make them more prone to microcracking and microcutting compared to zirconia. Finally, a positive correlation has been found between the surface roughness of zirconia and enamel wear [28].

An array of six global laboratory assessment methods has been described in the literature for the simulation and quantification of prosthetic material enamel wear [35]. Particularly, these constitute: the ACTA method [36], Alabama method [37], Ivoclar method [38], Munich method [39], UHSU method [40], and Zurich method [41]. Each method is characterized by a specific loading, frequency, and thermocycling protocol, or treatment of the material surface and type of outcome assessment. However, no specifically described protocols complying with the aforementioned methodologies have been followed by the primary studies included in this review. In essence, each followed a customized approach for simulation of masticatory cycle movements and assessment methodology. This was apparently a contributing factor towards the heterogeneity of the existing evidence, as well as the lack of standardization of the laboratory conditions in practice.

On the same grounds, a recent systematic review on wear diagnostics and reliability for dental materials and tissues [42] has pinpointed the imperative need for the standardization of assessment procedures and has given rise to certain methodological parameters that may contribute to the final outcome assessment. It has been apparent that accuracy considerations in isolation do not suffice, while other methodological aspects, such as the calibration procedures, the precision of scanning methodologies, and the comparable acquisition devices (i.e., profilometer) with matching software, are significant counterparts and should be considered if globally usable data are to be expected. Another source of potential bias identified by the literature is the problem of unusable data. While no reporting of missing data was recorded within the included studies of the present systematic review, there are claims that there is a non-negligible amount of data collected by researchers while conducting a laboratory or clinical project that does not ultimately contribute to the final outcome assessment or feed into the retrieved findings [43,44]. Some examples are the waste of impressions, unreliable scans, unexpected occlusal adjustments, and patient drop- outs. 

In vitro research, comprising of laboratory simulation studies of the masticatory or chewing function, constitute an initial tool for the comprehension of the clinical behavior of restorative materials in practice. Up to now, there has been a paucity of clinical studies on enamel wear and this was somewhat expected if one considers the potentially uncontrolled prognostic factors and the difficulties overshadowing such efforts. Enamel tooth wear directly related to antagonist prosthetic materials/restorations or natural dentition cannot be easily isolated. Parameters, such as mastication or chewing functional capacity and occlusal forces, or more extreme conditions, such as bruxism, abrasiveness of daily diet, or anatomical considerations of the dentition, should be considered when planning forthcoming research in vivo [2,3,45]. Therefore, well-designed controlled clinical trials of a randomized and split-mouth design to eliminate selection bias are welcomed, while laboratory research characterized by the use of standardized and consistent methodology protocols and core outcome sets [46] that may reduce heterogeneity issues will support this effort. 

### 4.2. Strengths and Limitations

This review presents several strengths. It is the first systematic review with meta-analysis and meta-regression on enamel tooth wear considering evidence from both pre-clinical (i.e., laboratory, in vitro) and clinical research. The review protocol was registered within two open repositories prior to formal initiation of the study. The search strategy was done through a comprehensive literature search within published and unpublished literature. Transparent methodologies used aimed for elimination of reporting and publication bias at the design stage. 

However, the review might have been prone to certain limitations. Methodological and risk of bias issues existed for all included studies from both in vitro and clinical research and this might have influenced the perceived findings of the study. All meta-analyses were based on a small number of studies (practically two) with small sample sizes and in some cases were prone to heterogeneity. This might have a bearing on the precision of the recorded estimates and the confidence in the estimated effect. Although sensitivity analyses were initially planned, we were not able to proceed with that, as there was a paucity of trials for inclusion in the quantitative synthesis. 

## 5. Conclusions

In light of the retrieved evidence stemming from the present systematic review, it may be confirmed that there is a great variability across prosthetic materials and their clinical or simulated behavior in relation to antagonist enamel wear. Some indicate the increased potential of certain materials for enamel wear of antagonist teeth and others highlight the between-material variations. Monolithic zirconia as well as conventional porcelain restorations showed evidence of increased wearing potential on the enamel. On the contrary, surface treatment, such as polishing of zirconia materials, was found to bear a less pronounced effect. In any case, it is evident that research on the topic should be guided by an increase in the number and quality of clinical studies that will promote and substantiate current knowledge from abundant laboratory research. 

## Figures and Tables

**Table 1:** Risk of bias of the included randomized trials (RoB 2.0 tool).

Reference	Randomization Process	Deviations from Intended Interventions	Mising Outcome Data	Measurement of the Outcome	Selection of the Reported Result	Overall
Esquivel- Upshaw 2018	**Low**	**Low**	**Low**	**Some** **concerns**	**Some** **concerns**	**Some concerns**
Mundhe 2015	**Some concerns**	**Low**	**Low**	**Some concerns**	**Some concerns**	**Some concerns**
Yang 2014	**Some concerns**	**Low**	**Low**	**Some concerns**	**Some concerns**	**Some concerns**

**Table 2:** Risk of bias of the included non-randomized studies (ROBINS-I tool).

	Bias due to / in…							
	Confounding	Selection of Participants into the Study	Classification of Interventions	Deviations from Intended Interventions	Missing Data	Measurement of Outcomes	Selection of the Reported Result	Overall
Stober 2014	**Moderate**	**No Information**	**Low**	**Low**	**Low**	**Moderate**	**Moderate**	**Moderate**

**Table 3:** Quantitative syntheses for enamel wear related to eligible outcomes/comparisons across in vitro and clinical studies.

#	Study ID	Outcome	Comparison	SMD (95% CIs)	*p*-value	Heterogeneity (I^2^ %)
**In vitro**						
**1**	**Two studies**	Volume wear (mm^3^)	E- PZ	1.06 (0.39, 1.73)	0.002	0
**2**	**Two studies**	Volume wear (mm^3^)	E- PGZ	–0.41 (–3.12, 2.30)	0.77	92.7
**3**	**Two studies**	Vertical wear (μm)	PZ- PGZ	0.0 (–0.59, 0.59)	0.99	0
**4**	**Two studies**	Volume wear (mm^3^)	PZ- PGZ	–3.03 (–9.20, 3.14)	0.34	95.4
**5**	**Two studies**	Volume wear (mm^3^)	PZ- GZ	–2.85 (–6.86, 1.15)	0.16	92.5
**6**	**Two studies**	Volume wear (mm^3^)	MZ- LD	–0.93 (–2.17, 0.32)	0.14	67.7
**7**	**Two studies**	Vertical wear (μm)	MZ- LD	–1.59 (–5.51, 2.33)	0.43	92.1
**8**	**Two studies**	Vertical wear (μm)	MZ- CR	0.36 (–0.39, 1.11)	0.34	0
**9**	**Two studies**	Vertical wear (μm)	LD- CR	1.84 (–1.97, 5.64)	0.34	91.1
**10**	**Two studies**	Vertical wear (mm)	CP- LFC	1.45 (–0.72, 3.62)	0.19	87.9
**11**	**Two studies**	Vertical wear (mm)	CP-MC	2.18 (1.34, 3.02)	<0.001	8.4
**12**	**Two studies**	Vertical wear (mm)	LFC- MC	0.34 (–1.7, 2.41)	0.75	89.5
**Clinical**						
	**Study ID**	**Outcome**	**Comparison**	**WMD (95% CIs)**	***p*-value**	**Heterogeneity (I^2^ %)**
**13**	**Two studies**	Vertical wear (μm)	MZ- E	107.38 (30.46, 184.30)	0.01	94.5

Minus (–) sign indicates greater enamel wear caused by the second presented material; SMD, standardized mean difference; CIs, confidence intervals; E, enamel; PZ, polished zirconia; PGZ, polished glazed zirconia; GZ, glazed zirconia; MZ, monolithic zirconia; LD, lithium disilicate; CR, composite resin; CP, conventional porcelain; LFC; low-fusing hydrothermal ceramic; MC, machinable ceramic; WMD, weighted mean difference.

## Supplementary Materials

The following are available online at https://www.mdpi.com/1996-1944/12/21/3575/s1, Table S1: Characteristics of included studies in alphabetical order (n = 27). Table S2: Detailed assessment of included randomized trials with the RoB 2.0 tool (supplement to Table 1). Table S3: Detailed assessment of included non-randomized studies with the ROBINS-I tool (supplement to Table 2).

## Appendix A

MEDLINE search 28 July 2019

Limits: no language restriction applied

Publication date: no restriction

Search Builder: ‘All Fields’

Four consecutive searches combined with “AND” Boolean operator, using “OR” between free text terms or keywords:

1. tooth wear

2. enamel wear

3. accelerat* tooth wear

4. dental wear

5. 1 OR 2 OR 3 OR 4 OR 5

6. function

7. mastication

8. cyclic

9. cycle*

10. chewing

11. 6 OR 7 OR 8 OR 9 OR 10

12. in vitro

13. in- vitro

14. simulate*

15. clinical

16. clinical condition*

17. in vivo

18. in- vivo

19. 12 OR 13 OR 14 OR 15 OR 16 OR 17 OR 18

20. resin

21. composite

22. porcelain

23. zirconia

24. enamel

25. ceramic

26. 20 OR 21 OR 22 OR 23 OR 24 OR 25

27. 5 AND 11 AND 19 AND 26
