# Supplementary material for: In Vitro Simulation and In Vivo Assessment of Tooth Wear: A Meta-Analysis of In Vitro and Clinical Research
Source: Materials (Basel). 2019 Oct 31;12(21):3575. doi: 10.3390/ma12213575 (PMC6862526; doi:10.3390/ma12213575)
Supplement: Supplementary file 1 [file materials-12-03575-s001.pdf]

materials

MDPI

Supplementary

In Vitro Simulation and In Vivo Assessment of Tooth Wear: A Meta-Analysis of In Vitro and Clinical Research

Despina Koletsi [1], Anna Iliadi [2], Theodore Eliades [1,*] and George Eliades [2]

[1] Clinic of Orthodontics and Pediatric Dentistry, Center of Dental Medicine, University of Zurich, 8032 Zurich, Switzerland; d.koletsi@gmail.com
[2] Department of Biomaterials, School of Dentistry, National and Kapodistrian University of Athens, 11527 Athens, Greece; annaeliades@gmail.com (A.I.); geliad@dent.uoa.gr (G.E.)
* Correspondence:Correspondence: Theodore.Eliades@zzm.uzh.ch

Table S1. Characteristics of included studies in alphabetical order (n = 27).

Author	Year	Origin	Design	Sample size/ enamel teeth type	Analysis Method	Groups under Comparison	Outcomes	Simulation Information/ follow-up
Aldegheishem [1]	2015	Germany	In vitro	21 molars, lingual crown sections	laser scanner	enamel (n = 7 each) vs different zirconia materials: 1. NanoZr (P-NANOZR, Panasonic Healthcare), 2. Zeno (Zenostar, Wieland), 3. Cercon (Cercon HT, DeguDent). Subgoroups (surface roughness): smooth (S) (Ra = 0.01 μm), moderate (M) (Ra = 0.1 μm), rough (R) (Ra = 1 μm)	1.volumetric wear	1,200,000 cycles thermomechanical fatigue, 98 N load, 1.6 Hz frequency (simulation of 5 years in vivo function)

Al-Hiyasat [2]	1997	Jordan	In vitro	30 mandibular premolars, 60 cusps	reflex microscope	enamel (n = 10 each) vs. 1. glazed porcelain, 2. unglazed porcelain, polished porcelain	1. mean wear depth	5000, 15,000, 25,000 cycles of wear testing, 40 N load
Al-Hiyasat [3]	1998	Jordan	In vitro	25 mandibular premolars, 50 cusps	reflex microscope	enamel (n = 10 each) vs. 1. aluminous porcelain Vitadur Alpha, 2. bonded to metal porcelain Omega, 3. Low-fusing hydrothermal ceramic Duceram-LFC, 4. machinable ceramic Vita Mark II, 5. cast type IV gold	1. mean wear	5000, 15,000, 25,000 cycles of wear testing, under a load of 40 N
Al-Hiyasat [4]	1999	Jordan	In vitro	15 mandibular premolars, 30 cusps	reflex microscope	enamel (n = 10 each) vs. 1. conventional porcelain, 2. Low-fusing hydrothermal ceramic Duceram-LFC, 3. machinable ceramic Vita Mark II	1. mean wear	5000, 15,000, 25,000 cycles of wear testing, under a load of 40 N
Ashtiani [5]	2019	Iran	In vitro	20 maxillary premolars	photography using a stereomicroscope (SF-100B, Lomo, Russia)	enamel (n = 10 each), vs. 1. feldspathic porcelain (VMK 95, Vita), 2. polymer-infiltrated ceramics (Vita Enamics)	1. tooth wear	120,000 cycles, chewing simulator, under a load of 5kg (~49 N) and 1.6 Hz frequency of antagonist movement
Bedini [6]	2012	Italy	In vitro	25 molars	fatigue test by means of the servo-hydraulic 858 MiniBionix, testing machine (MTS	enamel (n = 5 each), vs. 1. enamel, 2. hybrid microceramic composite, 3. PEX composite, 4. PFS composite	1. volumetric fatigue difference, 2. surface fatigue difference	1,000,000 cycles, compression load ranging from 34 to 340 N at a frequency of 6 Hz (i.e., this is a

					System Co., Minneapolis, MN, USA)			simulation of 5 years in vivo aging)
Chong [7]	2015	Australia	In vitro	60 maxillary and manibular premolars and molars	two body wear resistance tests	enamel (n = 12 each), vs. 1. enamel (maxillary incosor), 2. Lab-polished zirconia, 3. Lab-polished and glazed zirconia, 4. clinically adjusted zirconia, 5. clinically adjusted and repolished zirconia	1. vertical enamel loss, 2. volumetric enamel loss	120,000 cycles, chewing simulator, under a load of 5 kg (~49 N) and 1.6 Hz frequency of antagonist movement
Esquivel-Upshaw [8]	2018	Florida, US	clinical (RCT)	30 patients, single crowns	laserscanner	16 monolithic zirconia, 14 metal-ceramic, and corresponding enamel antagonists	1. volume loss, 2. wear, 3. maximum vertical loss	6 month and 1 year evaluation
Fathy [9]	2018	Egypt	In vitro	25 maxillary premolars	cycling wear testing, chewing simulator	enamel (n = 30), vs. 1. Zirconia-reinforced lithium silicate glass ceramic (Vita Suprinity)	1. weight loss, for different pH (none, 3, 7.2) and storage periods (24 h, 1 week)	150,000 cycles, chewing simulator, under a load of 5 kg (~49 N) and 1.6 Hz frequency of antagonist movement (i.e., this is a simulation of 1 year of in vivo aging)
Ghazal [10]	2008	Germany	In vitro	8 maxillary first premolars	custom made mechanical profilometer	enamel opposed to steatite ball (n = 8) and compared to: 1. Feldspathic ceramic, 2. composite resin, 3. IPN resin, 4. PMMA resin networks, 5. TCR resin,	1. vertical substance loss, 2. volume loss	120,000, 240,000, 480,000, 840,000, and 1,200,000 cycles, chewing simulator (steatite ball), under a load of 5 kg (~49 N) and 1.6 Hz frequency

						6. DCL resin, 7. PMMA derivative form		of antagonist movement
Ghazal [11]	2009	Germany	In vitro	24 maxillary first premolars	laser scanner	enamel opposed to zirconia ceramic balls of three different surface roughness (i.e., conventional, abraded with 50-µm alumina particles and 0.5 bar, and abraded with 50-µm alumina particles and 1 bar), compared to: 1. Nano-filled composite resin	1. vertical substance loss, 2. volume loss	300,000 cycles, mastication simulator, under a load of 5kg (~49 N) (zirconia ceramic ball)
Gundugollu [12]	2018	India	In vitro	60 maxillary first premolars	cycling wear testing, chewing simulator	enamel vs. 1. unpolished unglazed layered zirconia, 2. polished unglazed layered zirconia, 3. polished glazed layered zirconia, 4. unpolished unglazed monolithic zirconia, 5. polished unglazed monolithic zirconia, 6. polished glazed monolithic zirconia	1. vertical substance loss, 2. volume loss	250,000 cycles, under load of 5 kg (~49 N), (i.e., this is a simulation of 1-year in vivo chewing)
Habib [13]	2019	Saudi Arabia	In vitro	32 premolars	3D profilometer	enamel (8 each), vs. 1. monolithic zirconia, 2. lithium disilicate, 3. ceramic fused to metal, 4. composite resin	1. vertical height loss, 2. weight	240,000 cycles, under a load of 49 N, 0.8 Hz

Janyavula [14]	2013	Alabama, US	In vitro	8 mandibular molars	non contact surface profilometer	enamel vs. 1. polished zirconia, 2. glazed zirconia, 3. polished glazed zirconia, 4. veneering porcelain, 5. incisor enamel	1. volumetric wear	200,000 and 400,000 cycles, under a load of 10 N
Jung [15]	2010	Korea	In vitro	60 premolars	3D profilometer	enamel (n = 20 each) vs. 1. feldspathic porcelain, 2. zirconia polished, 3. zirconia glazed	1. volume loss	250,000 cycles, under load of 5 kg (~49 N), (i.e., this is a simulation of 1-year in vivo chewing)
Kim [16]	2012	Korea	In vitro	100 maxillary and mandibular premolars	MTS 3D profiler	enamel (n = 10 each) vs. 600 and 1200 grit of groups: 1. monolithic zirconia Pretteau, 2. monolithic zirconia Lava, 3. monolithic zirconia Rainbow, 4. lithium disilicate e.Max Press, 5. feldspathic porcelain Vita-Omega	1. volume loss	300,000 cycles, chewing simulator, under load of 5 kg (~49 N), (i.e., this is a simulation of 1-year in vivo chewing)
Lawson [17]	2014	Alabama, US	In vitro	64 molars	Non-contact surface profilometer	enamel (n = 8 each), vs. 1. adjusted zirconia LAVA, 2. adjusted polished zirconia LAVA, 3. adjusted glazed zirconia LAVA, 4. adjusted lithium disilicate e.Max, 5. adjusted polished lithium disilicate e.Max, 6. adjusted glazed	1. volume loss	300,000 cycles, chewing simulator, under load of 10 N

						lithium disilicate e.Max, 7. veneering porcelain Ceramco, 8. maxillary dental incisors enamel		
Lee [18]	2014	New Zealand	In vitro	5 premolars/10 cusps	wear testing apparatus	enamel (n = 5 each), vs. 1. lithium disilicate glass ceramic e.Max, 2. type III gold	1. friction coefficient for wear (μ)	1100 cycles, 9.8 N and 1.6 Hz
Mulay [19]	2015	India	In vitro	60 maxillary first premolars	weight testing machine	enamel (n = 15 each), vs. 1. autoglazed ceramic, 2. overglazed ceramic, 3. ceramic polished with Shofu kit, 4. ceramic polished with DFS wheels	1. percentage weight loss	5,000- 10,000 cycles
Mundhe [20]	2015	India	clinical (RCT)	10 patients, 30 teeth	3D scanner for casts	enamel (10 each), vs. 1. antagosinst enamel, 2. metal-ceramic, 3. monolithic zirconia	1. wear	1 year evaluation
Olivera [21]	2006	Brazil	In vitro	80 maxillary premolars	surface analyzer and computer program	enamel (n = 10 each), vs. 1. glazed ceramics, 2. polished ceramics of 5 commercial products (Empress 2, IPS, D-plus, D LFC, Symbio)	1. total enamel wear	150,000 and 300,000 cycles, under load 20 N, chewing rate 1.3 Hz
Rupawala [22]	2017	India	In vitro	60 maxillary first and second premolars	wear testing apparatus	enamel (n = 15 each), vs. 1. glazed zirconia, 2. polished unglazed zirconia, 3. porcelain fused to metal, 4. lithium disilicate	1. loss of height	10,000 cycles, under load of 5 kg (50 N)

Sripetchdanond [23]	2014	Thailand	In vitro	24 molars	profilometer	enamel (n = 6 each), vs. 1. monolithic zirconia, 2. lithium disilicate glass ceramic, 3. composite resin, 4. enamel of occlusan surface of third molar	1. maximum depth of wear, 2. mean depth of wear	4800 cycles, under load of 25 N
Stawarczyk [24]	2013	Switzerland	In vitro	30 maxillary molars	3D profilometer	enamel (n = 6 each), vs. 1. veneered zirconia, 2. glazed zirconia ceramic, 3. manually polished monolithic zirconia, 4. mechanically polished monolithic zirconia, 5. monolithic base alloy	1. enamel wear	120,000, 240,000, 640,000, and 1,200,000 masticatory cycles
Stober [25]	2014	Germany	clinical	17 participants (mean age 43, SD 14) with corresponding/ contralateral molar crowns	3D laser scanner	enamel (n = 17 each), vs. 1. monolithic zirconia, 2. enamel molar antagonist	1. mean vertical loss, 2. maximum vertical loss	6-month evaluation
Yang [26]	2019	Korea	invitro and clinical (RCT)	In vitro: 60 maxillary premolars; RCT: 30 patients requiring implant restoration of 1st/2nd molar	desktop scanner	enamel (n = 15 each), vs. 1. polished Rainbow zirconia, 2. polished Katania zirconia	1. vertical wear	In vitro: 100,000 cycles, under load of 5 kg (~49 N), (i.e., this is a simulation of 6 months in vivo chewing); RCT: 6-month evaluation
Zheng [27]	2016	China	In vitro	40 teeth/80 samples	laser scanning microscopy and 3D surface profilometer	enamel (n = 6 each) against silicon carbide ceramic ball, compared: 1. dried, 2. fresh enamel	1. wear volume	5000, 50,000, 250,000, 550,000, 800,000, and 1,000,000 cycles under load of 20 N

Table S2. Detailed assessment of the included randomized trials with the RoB 2.0 tool (supplement to Table 1).

Domain	Reference	Esquivel-Upshaw 2018	Mundhe 2015	Yang 2014
1. Randomization process	1.1	Y	Y	Y
	1.2	PY	NI	NI
	1.3	N	PN	PN
	Judgement	Low	Some concerns	Some concerns
2. Deviations from intended interventions	2.1	N	NI	NI
	2.2	PY	NI	NI
	2.3	PN	PN	PN
	2.4	NA	NA	NA
	2.5	NA	NA	NA
	2.6	PY	PY	PY
	2.7	NA	NA	NA
	Judgement	Low	Low	Low
3. Missing outcome data	3.1	Y	Y	Y
	3.2	NA	NA	NA
	3.3	NA	NA	NA
	3.4	NA	NA	NA
	Judgement	Low	Low	Low
4. Measurement of the outcome	4.1	N	N	N
	4.2	PN	N	N
	4.3	NI	NI	NI
	4.4	PY	PY	PY
	4.5	PN	PN	PN
	Judgement	Some concerns	Some concerns	Some concerns
5. Selection of the reported result	5.1	NI	NI	NI
	5.2	PN	PN	PN
	5.3	PN	PN	PN
	Judgement	Some concerns	Some concerns	Some concerns
Overall	Judgement	Some concerns	Some concerns	Some concerns

Y, yes; PY, probably yes; N, no; PN, probably no; NI, no information; NA, not applicable.

Table S3. Detailed assessment of the included non-randomized studies with the ROBINS-I tool (supplement to Table 2).

Domain	Reference	Stober 2014
1. Confounding	1.1	PY
	1.2	N
	1.3	N
	1.4	PY
	1.5	PY
	1.6	N
	1.7	NA
	1.8	NA
	Judgement	Moderate
2. Selection of participants into the study	2.1	NI
	2.2	NA
	2.3	NA
	2.4	NI
	2.5	NA
	Judgement	NI
3. Classification of interventions	3.1	Y
	3.2	Y
	3.3	PN
	Judgement	Low
4. Deviations from intended interventions	4.1	PN
	4.2	NA
	4.3	NA
	4.4	NA
	4.5	NA
	4.6	NA
	Judgement	Low
5. Missing data	5.1	PY
	5.2	N
	5.3	NI

	5.4	NA
	5.5	NA
	Judgement	Low
6. Measurement of outcomes	6.1	PY
	6.2	NI
	6.3	PY
	6.4	PN
	Judgement	Moderate
7. Selection of the reported result	7.1	NI
	7.2	PN
	7.3	PN
	Judgement	Moderate
Overall	Judgement	Moderate

Y, yes; PY, probably yes; N, no; PN, probably no; NI, no information; NA, not applicable.

References

1. Aldegheishem, A.; Alfaer, A.; Brezavšček, M.; Vach, K.; Eliades, G.; Att, W. Wear behavior of zirconia substrates against different antagonist materials. *Int J Esthet Dent* **2015**, *10*, 468–485.
2. al-Hiyasat, A.S.; Saunders, W.P.; Sharkey, S.W.; Smith, G.M.; Gilmour, W.H. The abrasive effect of glazed, unglazed, and polished porcelain on the wear of human enamel, and the influence of carbonated soft drinks on the rate of wear. *Int J Prosthodont* **1997**, *10*, 269–282.
3. Al-Hiyasat, A.S.; Saunders, W.P.; Sharkey, S.W.; Smith, G.McR.; Gilmour, W.H. Investigation of human enamel wear against four dental ceramics and gold. *Journal of Dentistry* **1998**, *26*, 487–495.
4. Al-Hiyasat, A.S.; Saunders, W.P.; Smith, G.M. Three-body wear associated with three ceramics and enamel. *The Journal of Prosthetic Dentistry* **1999**, *82*, 476–481.
5. Ashtiani, A.H.; Azizian, M.; Rohani, A. Comparison the degree of enamel wear behavior opposed to Polymer-infiltrated ceramic and feldspathic porcelain. 7.
6. Bedini, R.; Pecci, R.; Notarangelo, G.; Zuppante, F.; Persico, S.; Carlo, F.D. Microtomography evaluation of dental tissue wear surface induced by in vitro simulated chewing cycles on human and composite teeth. *Annali dell'Istituto Superiore di Sanità* **2012**.
7. Chong, B.J.; Thangavel, A.K.; Rolton, S.B.; Guazzato, M.; Klineberg, I.J. Clinical and laboratory surface finishing procedures for zirconia on opposing human enamel wear: A laboratory study. *J Mech Behav Biomed Mater* **2015**, *50*, 93–103.
8. Esquivel-Upshaw, J.F.; Kim, M.J.; Hsu, S.M.; Abdulhameed, N.; Jenkins, R.; Neal, D.; Ren, F.; Clark, A.E. Randomized clinical study of wear of enamel antagonists against polished monolithic zirconia crowns. *Journal of Dentistry* **2018**, *68*, 19–27.
9. Fathy, S.M.; Swain, M.V. In-vitro wear of natural tooth surface opposed with zirconia reinforced lithium silicate glass ceramic after accelerated ageing. *Dental Materials* **2018**, *34*, 551–559.
10. Ghazal, M.; Yang, B.; Ludwig, K.; Kern, M. Two-body wear of resin and ceramic denture teeth in comparison to human enamel. *Dental Materials* **2008**, *24*, 502–507.
11. Ghazal, M.; Kern, M. The influence of antagonistic surface roughness on the wear of human enamel and nanofilled composite resin artificial teeth. *The Journal of Prosthetic Dentistry* **2009**, *101*, 342–349.
12. Gundugollu, Y.; Yalavarthy, R.; Krishna, Mh.; Kalluri, S.; Pydi, S.; Tedlapu, S. Comparison of the effect of monolithic and layered zirconia on natural teeth wear: An in vitro study. *J Indian Prosthodont Soc* **2018**, *18*, 336.
13. Habib, S.R.; Alotaibi, A.; Al Hazza, N.; Allam, Y.; AlGhazi, M. Two-body wear behavior of human enamel versus monolithic zirconia, lithium disilicate, ceramometal and composite resin. *J Adv Prosthodont* **2019**, *11*, 23.
14. Janyavula, S.; Lawson, N.; Cakir, D.; Beck, P.; Ramp, L.C.; Burgess, J.O. The wear of polished and glazed zirconia against enamel. *The Journal of Prosthetic Dentistry* **2013**, *109*, 22–29.
15. Jung, Y.-S.; Lee, J.-W.; Choi, Y.-J.; Ahn, J.-S.; Shin, S.-W.; Huh, J.-B. A study on the *in-vitro* wear of the natural tooth structure by opposing zirconia or dental porcelain. *J Adv Prosthodont* **2010**, *2*, 111.
16. Kim, M.-J.; Oh, S.-H.; Kim, J.-H.; Ju, S.-W.; Seo, D.-G.; Jun, S.-H.; Ahn, J.-S.; Ryu, J.-J. Wear evaluation of the human enamel opposing different Y-TZP dental ceramics and other porcelains. *Journal of Dentistry* **2012**, *40*, 979–988.
17. Lawson, N.C.; Janyavula, S.; Syklawer, S.; McLaren, E.A.; Burgess, J.O. Wear of enamel opposing zirconia and lithium disilicate after adjustment, polishing and glazing. *Journal of Dentistry* **2014**, *42*, 1586–1591.
18. Lee, A.; Swain, M.; He, L.; Lyons, K. Wear behavior of human enamel against lithium disilicate glass ceramic and type III gold. *The Journal of Prosthetic Dentistry* **2014**, *112*, 1399–1405.
19. 19. Mulay, G.; Dugal, R.; Buhranpurwala, M. An evaluation of wear of human enamel opposed by ceramics of different surface finishes. *J Indian Prosthodont Soc* **2015**, *15*, 111–118.
20. Mundhe, K.; Jain, V.; Pruthi, G.; Shah, N. Clinical study to evaluate the wear of natural enamel antagonist to zirconia and metal ceramic crowns. *The Journal of Prosthetic Dentistry* **2015**, *114*, 358–363.
21. Olivera, A.B.; Matson, E.; Marques, M.M. The Effect of Glazed and Polished Ceramics on Human Enamel Wear. 2.

22. Rupawala, A.; Musani, S.I.; Madanshetty, P.; Dugal, R.; Shah, U.D.; Sheth, E.J. A study on the wear of enamel caused by monolithic zirconia and the subsequent phase transformation compared to two other ceramic systems. *J Indian Prosthodont Soc* **2017**, *17*, 8–14.
23. Sripetchdanond, J.; Leevailoj, C. Wear of human enamel opposing monolithic zirconia, glass ceramic, and composite resin: An in vitro study. *The Journal of Prosthetic Dentistry* **2014**, *112*, 1141–1150.
24. Stawarczyk, B.; Özcan, M.; Schmutz, F.; Trottmann, A.; Roos, M.; Hämmerle, C.H.F. Two-body wear of monolithic, veneered and glazed zirconia and their corresponding enamel antagonists. *Acta Odontologica Scandinavica* **2013**, *71*, 102–112.
25. Stober, T.; Bermejo, J.L.; Rammelsberg, P.; Schmitter, M. Enamel wear caused by monolithic zirconia crowns after 6 months of clinical use. *J Oral Rehabil* **2014**, *41*, 314–322.
26. Yang, S.-W.; Kim, J.-E.; Shin, Y.; Shim, J.-S.; Kim, J.-H. Enamel wear and aging of translucent zirconias: In vitro and clinical studies. *J Prosthet Dent* **2019**, *121*, 417–425.
27. Zheng, J.; Zeng, Y.; Wen, J.; Zheng, L.; Zhou, Z. Impact wear behavior of human tooth enamel under simulated chewing conditions. *Journal of the Mechanical Behavior of Biomedical Materials* **2016**, *62*, 119–127.

© 2019 by the authors. Submitted for possible open access publication under the terms and conditions of the Creative Commons Attribution (CC BY) license (http://creativecommons.org/licenses/by/4.0/).
